# Spike Protein of SARS-CoV-2 Activates Cardiac Fibrogenesis through NLRP3 Inflammasomes and NF-κB Signaling

**DOI:** 10.3390/cells13161331

**Published:** 2024-08-11

**Authors:** Huynh Van Tin, Lekha Rethi, Satoshi Higa, Yu-Hsun Kao, Yi-Jen Chen

**Affiliations:** 1 International Ph.D. Program in Medicine, College of Medicine, Taipei Medical University, Taipei 11031, Taiwan; d142109010@tmu.edu.tw; 2 Department of Orthopedics, Shuangho Hospital, Taipei Medical University, Taipei 11031, Taiwan; lekha@tmu.edu.tw; 3 Cardiac Electrophysiology and Pacing Laboratory, Division of Cardiovascular Medicine, Makiminato Central Hospital, Okinawa 901-2131, Japan; higa@haku-ai.or.jp; 4 Graduate Institute of Clinical Medicine, College of Medicine, Taipei Medical University, Taipei 11031, Taiwan; 5 Department of Medical Education and Research, Wan Fang Hospital, Taipei Medical University, Taipei 11031, Taiwan; 6 Division of Cardiovascular Medicine, Department of Internal Medicine, Wan Fang Hospital, Taipei Medical University, Taipei 11031, Taiwan

**Keywords:** angiotensin-converting enzyme 2, cardiac fibroblasts, NLRP3 inflammasome, spike protein

## Abstract

Background: The spike protein of severe acute respiratory syndrome coronavirus 2 (SARS-CoV-2) is crucial to viral entry and can cause cardiac injuries. Toll-like receptor 4 (TLR4) and NOD-, LPR-, and pyrin-domain-containing 3 (NLRP3) inflammasome are critical immune system components implicated in cardiac fibrosis. The spike protein activates NLRP3 inflammasome through TLR4 or angiotensin-converting enzyme 2 (ACE2) receptors, damaging various organs. However, the role of spike protein in cardiac fibrosis in humans, as well as its interactions with NLRP3 inflammasomes and TLR4, remain poorly understood. Methods: We utilized scratch assays, Western blotting, and immunofluorescence to evaluate the migration, fibrosis signaling, mitochondrial calcium levels, reactive oxygen species (ROS) production, and cell morphology of cultured human cardiac fibroblasts (CFs) treated with spike (S1) protein for 24 h with or without an anti-ACE2 neutralizing antibody, a TLR4 blocker, or an NLRP3 inhibitor. Results: S1 protein enhanced CFs migration and the expressions of collagen 1, α-smooth muscle actin, transforming growth factor β1 (TGF-β1), phosphorylated SMAD2/3, interleukin 1β (IL-1β), and nuclear factor kappa-light-chain-enhancer of activated B cells (NF-κB). S1 protein increased ROS production but did not affect mitochondrial calcium content and cell morphology. Treatment with an anti-ACE2 neutralizing antibody attenuated the effects of S1 protein on collagen 1 and TGF-β1 expressions. Moreover, NLRP3 (MCC950) and NF-kB inhibitors, but not the TLR4 inhibitor TAK-242, prevented the S1 protein-enhanced CFs migration and overexpression of collagen 1, TGF-β1, and IL-1β. Conclusion: S1 protein activates human CFs by priming NLRP3 inflammasomes through NF-κB signaling in an ACE2-dependent manner.

## 1. Introduction

The COVID-19 pandemic has had a devastating worldwide impact, and many individuals with a history of COVID-19 have cardiovascular complications [1,2,3,4]. One cardiovascular complication of particular concern is cardiac fibrosis, which adversely affects cardiac function and increases overall morbidity and mortality rates [5,6,7]. Several studies have underscored the role of the spike protein of SARS-CoV-2 in influencing cardiovascular pathology [8,9,10,11,12,13,14,15,16]. Notably, one study specifically reveals that the SARS-CoV-2 spike protein induces cardiac fibrosis by causing long-term transcriptional suppression of mitochondrial metabolic genes in obese mice, leading to impaired myocardial contractility and increased cardiac fibrosis, as shown using a spike protein pseudotyped virus model. This contributes to long COVID-related cardiomyopathy [9]. However, the mechanisms underlying this pro-fibrotic effect remain unknown. These findings prompt us to further investigate the molecular mechanisms by which the spike protein activates cardiac fibrosis and to identify potential therapeutic targets to mitigate this condition.

The spike protein of SARS-CoV-2 interact with angiotensin-converting enzyme 2 (ACE2) receptors [17,18]. ACE2 is expressed in respiratory epithelial cells and various cell types within the cardiovascular system, such as cardiomyocytes and CFs [19,20,21,22]. The interaction of ACE2 receptors and the spike protein of SARS-CoV-2 facilitates viral entry into host cells and initiates a cascade of intracellular events that may cause cardiovascular dysfunction. Furthermore, the spike protein activates CFs, causing phenotypic transformation and promoting fibrotic processes [9]. However, the precise mechanisms underlying spike protein-induced activation of CFs and the implications of this activation for cardiac fibrosis remain poorly understood.

The NOD-, LPR-, and pyrin-domain-containing 3 (NLRP3) inflammasome and toll-like receptor 4 (TLR4) are critical components of the immune system in the body’s defense against pathogens such as viruses [23,24]. The NLRP3 inflammasome and TLR4 recognize pathogen-associated molecular patterns (PAMPs) and damage-associated molecular patterns (DAMPs) and activate inflammatory responses [25,26]. Research reveals that spike protein can activate NLRP3 inflammasomes in lipopolysaccharide-primed microglia in an ACE2-dependent manner through nuclear factor kappa-light-chain-enhancer of activated B cell (NF-κB) signaling [27]. Furthermore, spike protein-induced TLR4 activation can trigger the activation of NLRP3 inflammasomes [28,29,30], suggesting that modulating the activity of NLRP3 and TLR4 might mitigate the inflammatory response and associated cardiac injury prompted by the SARS-CoV-2 spike protein. Accordingly, this study investigates the impacts of the SARS-CoV-2 spike protein on CFs and elucidates the molecular mechanisms underlying spike protein-induced fibrogenesis.

## 2. Materials and Methods

### 2.1. Human CFs Cultures

Human atrial CFs (NHCF-A) (Lonza, Basel, Switzerland, LONCC-2903) were cultured in Fibroblast Growth Medium-3 (FGM3, Lonza, CC-4526). Passage 5 NHCF-A cells were treated with or without 5 nM of S1 protein (Genscript Biotech, Piscataway, NJ, USA, Z03501) for 24 h in the presence or absence of 1 µM TAK-242 (a TLR-4 blocker, Millipore, Darmstadt, Germany, 614316), 10 µM MCC950 (an NLRP3 inhibitor, Sigma, St. Louis, MO, USA, CP-456773), 3 µM Bay 11-7802 (an NF-κB inhibitor, MCE, HY-13453), and 5 µM anti-ACE2 antibody (Sino Biological, Beijing, China, 10108-MM37) for the following experiments.

### 2.2. Scratch Assay

The CFs were cultured in 6-well plates in serum-free medium, and scratches were made after treatment with or without S1 protein for 24 h under the presence or absence of MCC950 (10 µM) and TAK-242 (1 µM). Images were taken immediately after making the scratch lines (0 h) and again after 12 h. The percentage of closure areas was calculated by dividing the difference between the areas at 0 h and 12 h by the areas of the scratches at 0 h.

### 2.3. MTS Proliferation Assay

CellTiter 96 AQueous One Solution Cell Proliferation Assay (MTS; Promega Corporation, Australia, G358C) was employed to study the effects of S1 protein on CFs proliferation following the manufacturer’s protocol.

### 2.4. Immunoblotting 

Proteins from CFs were extracted using a mammalian protein extraction reagent with protease and phosphatase inhibitors (Thermo Fisher Scientific, Waltham, MA, USA). Total proteins (30 µg) were separated using sodium dodecyl sulfate-polyacrylamide gel electrophoresis and then transferred to polyvinylidene difluoride membranes. The blocking membranes were incubated overnight with primary antibodies against the following targets: ACE2 (Abcam, Cambridge, UK, catalog no. ab15348), alpha-smooth-muscle actin (α-SMA) (ab32575, Abcam), collagen type 1 alpha 1 (COL1A1, sc-293182, Santa Cruz, CA, USA), transforming growth factor β1 (TGF-β1, 3711s, Cell Signaling, Danvers, MA, USA), phosphorylated SMAD2/3 (#8828, Cell Signaling), interleukin (IL)-1β (ab9722, Abcam), and phosphorylated-NFκB p65 (Ser536) (#3033, Cell Signaling). Secondary antibodies were added, and the membranes were incubated for 2 h at room temperature after being washed five times on an orbital shaker. Bound antibodies were detected using an enhanced chemiluminescence detection system (Millipore, Darmstadt, Germany). AlphaEase FC (Alpha Innotech, San Leandro, CA, USA) was used to analyze band intensities. The target bands were initially normalized to the internal control, glyceraldehyde-3-phosphate dehydrogenase (MBL, Nagoya, Japan) and then further normalized to the control.

### 2.5. TGF-β1 Enzyme-Linked Immunosorbent Assay (ELISA)

The secreted TGF-β in CFs-cultured medium was quantified using a Quantikine ELISA kit (DB100C, R&D Systems, Minneapolis, MN, USA) following the manufacturer’s instructions.

### 2.6. TGF-β1 Real-Time Polymerase Chain Reaction (RT-PCR)

CFs cultured in 6-well plates were treated with or without S1 protein for 24 h and total RNA was extracted using Trizol reagent (Invitrogen, 15596026) following the manufacturer’s protocol. Cells were lysed by adding 1 mL of Trizol reagent and incubated for 5 min at room temperature. The lysate was then transferred into 1.5 mL Eppendorf tubes, and 0.2 mL of chloroform was added, thoroughly mixed, and centrifuged at 12,000× *g* for 15 min at 4 °C. Subsequently, the aqueous phase was transferred to a new Eppendorf tube. RNAs were precipitated by adding 0.5 mL of isopropanol to the aqueous phase and incubated for 10 min at 4 °C, followed by centrifugation at 12,000× *g* at 4 °C. The RNA pellets were collected by discarding the supernatant, after which 1 mL of 75% ethanol was added, followed by brief vortexing and centrifugation for 5 min at 7500× *g* at 4 °C. Finally, the supernatant was discarded, and the pellets were air dried for 5 to 10 min, then resuspended in 20 µL of RNAase-free water.

RNA concentrations were determined using a nanodrop. Reverse transcription and qPCR were performed using the ReverTra Ace TM qPCR-RT kit (TOYOBO, Osaka, Japan, FSQ-101) and SYBR Green Real-Time PCR Master Mix (TOYOBO, Osaka, Japan, QPK-201) according to the manufacturer’s instructions. The primer sequences for TGF-β1 were forward 5′-TACCTGAACCCGTGTTGCTCTC-3′ and reverse 5′-GTTGCTGAGGTATCGCCAGGAA-3′. The relative changes in TGF-β1 transcript level were assessed by analyzing the threshold cycle (Ct) value and normalizing it to the respective Ct value of β-actin, followed by normalization to the control group. 

### 2.7. Statistical Analysis

Continuous variables are expressed as means ± standard errors. The comparison between S1 protein-treated and control cells was conducted using paired *t*-tests and a one-way repeated-measures analysis of variance followed by Tukey’s post hoc correction for multiple group comparisons. *p*-values of ≤ 0.05 was considered statistically significant.

## 3. Results 

### 3.1. Effects of S1 Protein on CFs Migration and Proliferation

**Figure 1:**
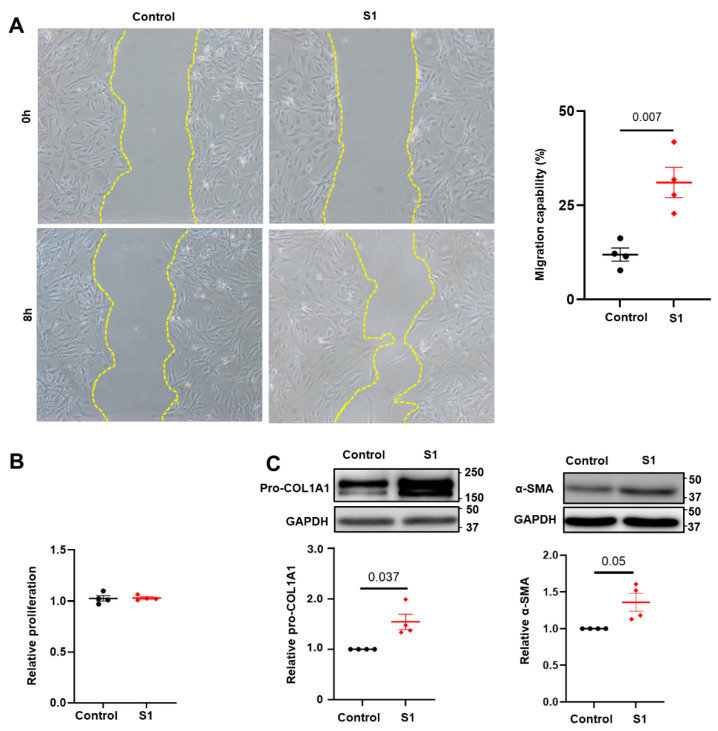
S1 protein enhanced CFs activation. Treatment with S1 protein (5 nM) for 24 h increased cell migration (**A**) but not cell proliferation (**B**) in CFs. (**C**) Additionally, S1 protein also elevated pro-COL1A1 and α-SMA protein expressions. *n* = 4 independent experiments.

To assess the impacts of S1 protein on CFs activation, we conducted a scratch assay and evaluated the expression of myofibroblast markers. Our findings revealed that S1 protein enhanced CFs migration ability (Figure 1A) and increased the expression of pro-COL1A1 and α-SMA (Figure 1C), while not affecting CFs proliferation (Figure 1B). These results indicate that S1 protein activates CFs.

### 3.2. Effects of S1 Protein on CFs Fibrosis Signaling 

**Figure 2:**
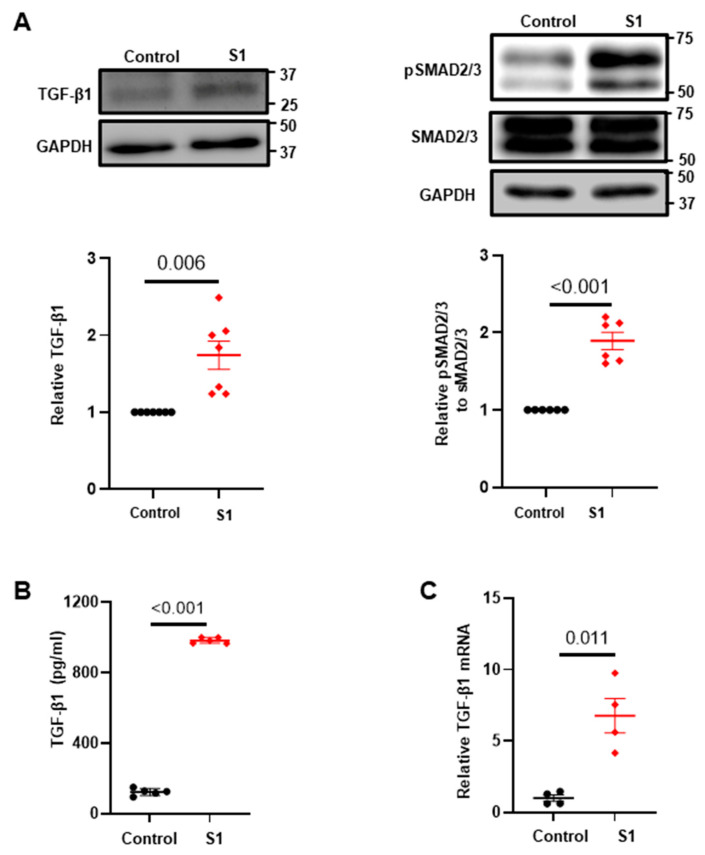
S1 protein increased profibrotic signaling. Treatment with S1 protein (5 nM) for 24 h increased protein expressions of TGF-β1 and pSMAD2/3 measured using Immunoblot ((**A**) *n* = 4 independent experiments), secretion of TGF-β1 in cultured medium measured using ELISA assays ((**B**) *n* = 5 independent experiments), and TGF-β1 mRNA quantified using RT-qPCR in CFs ((**C**) *n* = 4 independent experiments).

To elucidate the mechanisms underlying CFs activation by the S1 protein, we analyzed TGF-β1 expression at the protein and mRNA levels. Our results revealed that the S1 protein promoted TGF-β1 protein synthesis (Figure 2A), secretion (Figure 2B), and transcription (Figure 2C). Furthermore, S1 protein enhanced the expression of phosphorylated SMAD2/3, the downstream targets of TGF-β1 (Figure 2A), indicating that S1 protein directly influences the expression of extracellular matrix proteins by enhancing the fibrotic signaling.

### 3.3. Role of NLRP3 and TLR4 in S1 Protein-Induced CFs Activation

**Figure 3:**
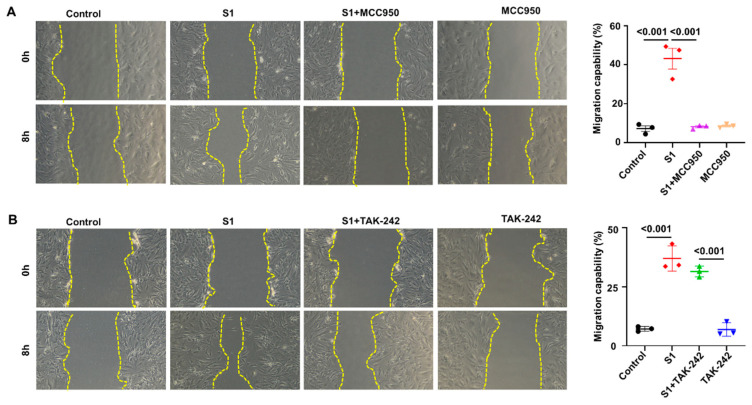
Impact of NLRP3 and TLR4 signaling on S1 protein-mediated CFs migration. Pretreatment with MCC950 (10 µM (**A**)) but not TAK-242 (1 µM (**B**)) blocked the effects of S1 protein (5 nM) treatment for 24 h on cell migration. *n* = 3 independent experiments.

**Figure 4:**
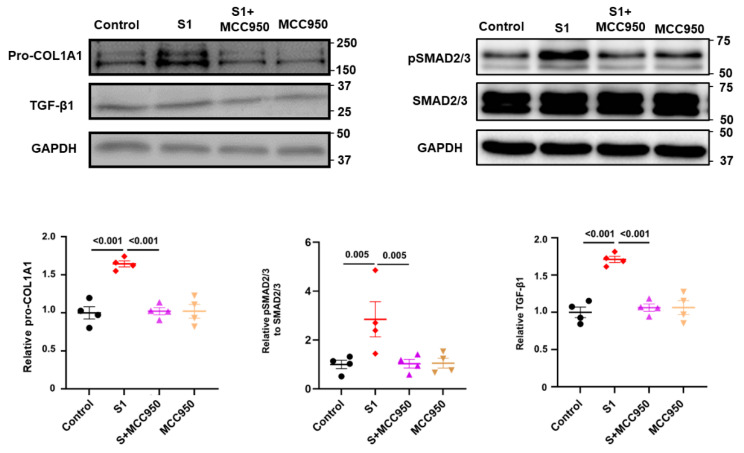
The role of NLRP3 signaling on S1 protein-induced fibrotic markers in CFs. MCC950 effectively blocked the effect of S1 on expressions of fibrotic markers including pro-COL1A1, TGF-β1, and its downstream target, pSMAD2/3. *n* = 4 independent experiments.

**Figure 5:**
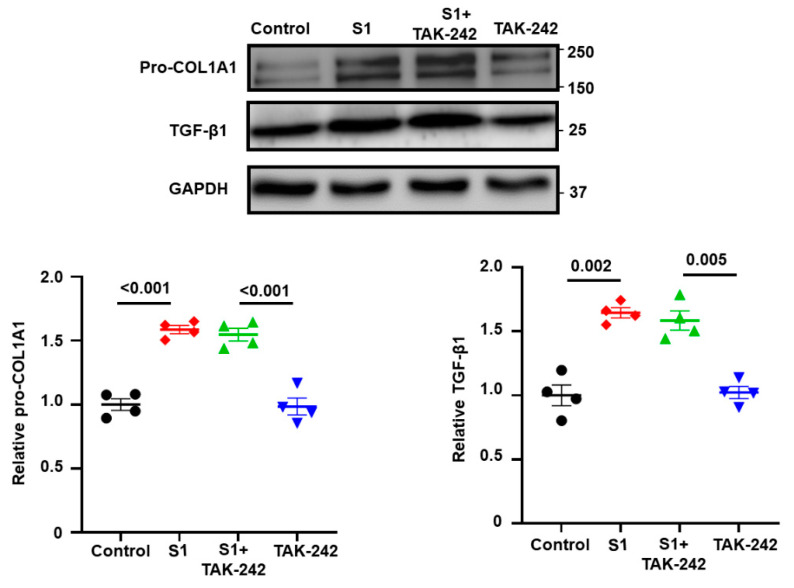
Role of TLR4 signaling on S1 protein-induced fibrotic markers in CFs. TAK-242 did not change the effect of S1 protein on pro-COL1A1 and TGF-β1 expressions in CFs (10 µM, B). *n* = 4 independent experiments.

To investigate the roles of NLRP3 and TLR4 in the activation of CFs by S1 protein, we pretreated CFs with MCC950 (an NLRP3 inhibitor) at 10 µM and TAK-242 (a TLR4 inhibitor) at 1 µM. We observed that MCC950 inhibited the effects of S1 protein on CFs migration (Figure 3A) and the expression of pro-COL1A1, TGF-β1, and pSMAD2/3 (Figure 4), while TAK-242 did not exhibit inhibitory effects on these processes (Figure 3B and Figure 5). 

### 3.4. Role of NF-kB Signaling in S1 Protein-Induced CFs Activation

**Figure 6:**
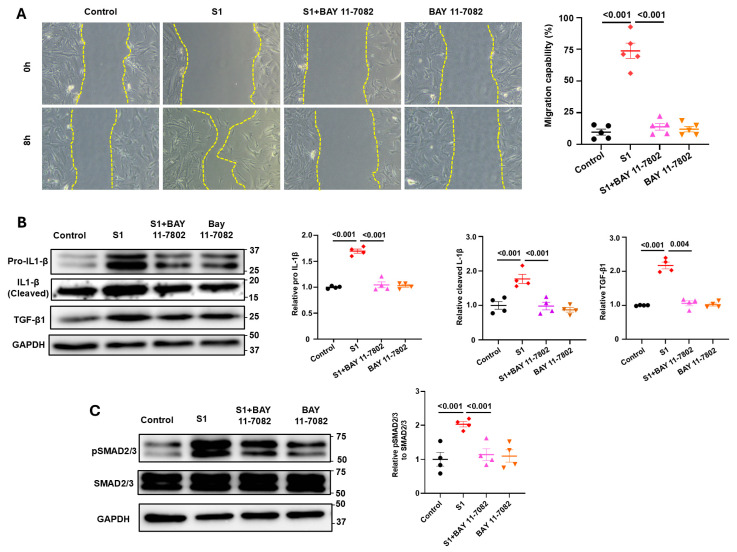
Role of NF-κB on S1 protein-mediated CFs migration and expressions of fibrotic factors. Pretreatment with BAY 11-7082 (an NF-κB inhibitor, 3 µM) completely blocked the effects of S1 protein (5 nM for 24 h) on CFs migration ((**A**) *n* = 5 independent experiments) and the protein expressions of IL1-β cleavage, TGF-β1, and pSMAD2/3 ((**B**,**C**) *n* = 4 independent experiments).

To investigate the involvement of NF-κB on the activation of CFs by the S1 protein, we pretreated CFs with Bay 11-7082 (3 µM), which is an NF-κB inhibitor, and discovered that Bay 11-7082 attenuated the effects of S1 protein on CFs migration (Figure 6A) and the expression of IL1-β and TGF-β (Figure 6B). This result indicates that NF-κB mediates the CFs activation effects induced by the S1 protein. Furthermore, we also checked the activation of IL-1β, which is a marker of NF-κB and NLRP3 inflammasome activation. Results showed that S1 protein increased IL-1β cleavage in CFs, and this effect was blocked by BAY 11-7802 (Figure 6B). Moreover, BAY 11-7082 also reversed the effect of the S1 protein on SMAD2/3 phosphorylation (Figure 6C).

### 3.5. S1 Protein Activates CFs in an ACE2-Dependent Manner

**Figure 7:**
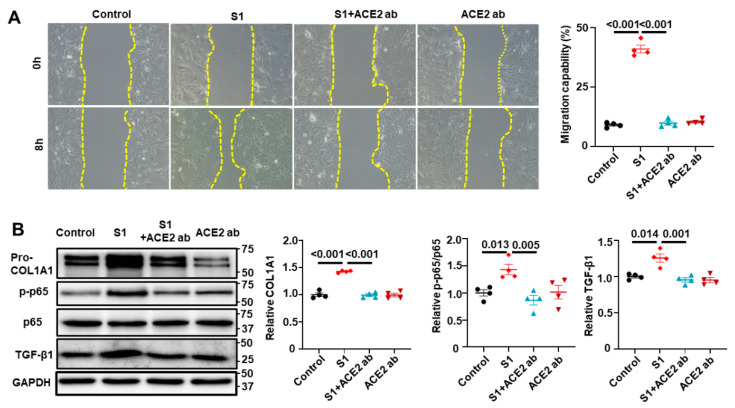
S1 protein activated CFs in an ACE2-dependent manner. ACE2 neutralization antibody (5 µM) effectively blocked the enhanced CFs migration induced by the S1 protein (5 nM for 24 h) (**A**), along with suppressing the protein expressions of pro-COL1A1, TGF-β1, and phosphorylated NF-κB (p-p65) (**B**). *n* = 4 independent experiments.

To investigate whether ACE2 receptors mediate the effects of S1 protein on CFs, we pretreated the cells with an anti-ACE2 antibody before adding S1 protein. Remarkably, in the presence of the anti-ACE2 antibody, the S1 protein did not affect the migration of CFs (Figure 7A) or the expression of pro-COL1A1 or TGF-β in CFs (Figure 7B). Moreover, S1 protein alone increased p-p65; however, this change was abolished by anti-ACE2 ab (Figure 7B). These results indicate that the S1 protein exerts its effects in an ACE2-dependent manner. 

## 4. Discussion

Since the first cases were identified in December 2019 [31], SARS-CoV-2 has rapidly spread worldwide and remains a global public health concern. As of 24 March 2024, 757,132,086 confirmed infections and 7,042,222 deaths have been reported [32]. The disease primarily affects the epithelial compartment in the upper and lower airways but also damages several organs, including the heart [33,34]. The incidence of cardiac injury in individuals with COVID-19 ranges from 7.2% to 19.7% [34]. Additionally, one study reported the persistence of COVID-19 symptoms in various systems beyond the acute phase [35], a condition known as post-acute COVID-19 syndrome. Cardiovascular complications, including myocardial injury, heart failure, arrhythmias, and coagulation disorders, manifest not only during the acute phase but also beyond the initial 30 days of SARS-CoV-2 infection, escalating mortality and morbidity [36]. However, the precise mechanisms underlying cardiac involvement in COVID-19 remain unclear. The spike protein of SARS-CoV-2, composed of subunits S1 and S2, plays a critical role in receptor recognition and cell membrane fusion during the viral cell entry process. The S1 protein binds directly to the ACE2 receptor, which is highly expressed in the heart, initiating the cell entry process [18]. Thus, spike protein may be implicated in cardiac complications in COVID-19. Evidence indicates that the spike protein can independently induce cardiovascular complications by binding to cell membrane receptors, leading to inflammation and damage to cells, tissues, and organs [37,38]. However, most studies have focused on the effects of spike protein on cardiomyocytes, the myocardium, cardiac pericytes, or vascular endothelial cells. Fibroblasts, among the most abundant cell types in the heart, are crucial to cardiac fibrosis, which contributes cardiac remodeling, arrhythmias, and heart failure [39] under pathological stimuli such as inflammatory cytokines, ROSs, TGF-β, and the renin–angiotensin–aldosterone system [40]. In this study, we used atrial fibroblasts because they exhibit greater secretory activity and reactivity, making them more suitable for studying the mechanisms of cardiac fibrosis. We discovered that exposure to the S1 protein significantly enhanced human CFs migration and induced the expression of key fibrotic markers, including COL1A1 and α-SMA, TGF-β1 and pSMAD2/3, and IL-1β. These results indicate that the S1 protein plays a crucial role in promoting fibrotic processes within cardiac tissues. Moreover, in an animal model study, Cao et al. discovered that spike protein promotes cardiac fibrosis in obese mice [9]. While they proposed that the spike protein causes myocardial contractile impairment in obese mice by inducing long-term aberrances in the cardiac transcriptional signatures of mitochondrial respiratory chain genes, such as ATP synthases, nicotinamide adenine dinucleotide:ubiquinone oxidoreductase, and cytochrome c oxidase gene members [9], they did not delineate the mechanism by which spike protein promotes cardiac fibrosis.

TLR4, a pattern recognition receptor, plays a pivotal role in the immune response to bacterial lipopolysaccharides and various endogenous and exogenous danger signals. In the heart, TLR4 signaling is activated by various DAMPs, eliciting downstream signaling pathways, including the NF-κB and MAPK pathways, which stimulate the production of proinflammatory cytokines and chemokines [41]. TLR4 activation has been associated with inflammatory and fibrotic responses. NLRP3 inflammasomes—multiprotein complexes—are components of intracellular pattern recognition receptors primarily responsible for detecting PAMPs and DAMPs within cells. They process and activate proinflammatory cytokines, such as IL-1β and IL-18 [42]. NLRP3 is one of the most extensively studied members within the human NLR family, which consists of 22 members. In cardiac fibrosis, various stimuli, including oxidative stress, mitochondrial dysfunction, and metabolic disturbances common in heart diseases, can activate NLRP3 inflammasomes. TLR4 and NLRP3 inflammasomes, critical components of the immune system, are highly involved in the development of cardiac fibrosis in response to inflammatory signals and pathogens. Previous studies have revealed that spike protein directly interacts with and activates TLR4 and its downstream signaling, triggering inflammatory responses in immune cells [29], the lungs [43], and the brain [44]. However, the results of the present study indicate that the TLR4 inhibitor (TAK-242) does not inhibit the effects of the S1 protein on CFs. Additionally, spike protein increases mitochondrial ROS production, leading to subsequent mitophagy blockage, NLRP3 activation, enhanced NF-κB activity, elevated IL-18 levels, impaired cardiopulmonary function, and cardiac fibrosis in human-ACE2 transgenic mice [45]. Albornoz et al. demonstrated that spike protein activates NLRP3 inflammasomes through NF-κB signaling in an ACE2-dependent manner [27]. Consistent with these results, our study showed that the NLRP3 inhibitor (MCC950) completely inhibited the effects of the S1 protein on CFs. Moreover, inhibition of the NF-κB signaling pathway effectively prevented CFs migration and suppressed the increase in pro-COL1A1, TGF-β1, and IL-1β expression induced by the S1 protein. These findings underscore the crucial roles of the NF-κB and NLRP3 inflammasome signaling pathways in mediating the profibrotic effects of the S1 protein on CFs, implicating the NF-κB signaling pathway in the cellular response to SARS-CoV-2 infection. Given the well-established role of NF-κB in regulating inflammatory and fibrotic processes, our findings suggest that targeting this pathway may offer promising therapeutic approaches for preventing or treating COVID-19-associated cardiac fibrosis.

Our previous study revealed that the S1 protein altered the biogenetics of human cardiomyocytes by inducing mitochondrial calcium overload, ROS accumulation, and impairing mitochondrial dynamics [10]. In the present study, although the S1 protein did not affect mitochondrial morphology (Figure S1) or mitochondrial calcium content (Figure S2), it did increase ROS production (Figure S3). Since ROS also activates NLRP3 inflammasomes, increased ROS levels promote IL-1β and TGF-β production, leading to the fibrotic effects observed in human CFs.

The present study revealed that treatment with an anti-ACE2 antibody effectively countered the effects of S1 protein on the expression of pro-COL1A1 and TGF-β1 in CFs. This finding is consistent with our previous study, which showed that the S1 protein induces dysfunction in human cardiomyocyte via ACE2 [10]. These findings underscore the role of the ACE2 receptor in mediating the cellular response to S1 protein and indicate a potential therapeutic strategy for mitigating SARS-CoV-2-induced cardiac fibrosis.

**Figure 8:**
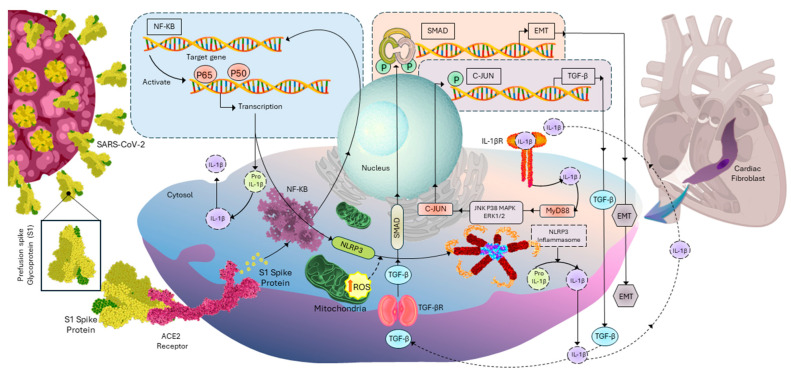
Mechanisms underlying S1 protein-induced activation of CFs and promotion of ECM protein synthesis. Key processes involve activation of the NLRP3 inflammasome, ACE2/NF-κB signaling, and ROS formation. Upon binding to ACE2, the S1 protein initiates a signaling cascade that activates NF-κB, a transcription factor promoting the expression of inflammation-related genes, including those required for NLRP3 inflammasome and pro-interleukin (IL)-1β. ROS formation triggers NLRP3 inflammasome activation, leading to processing of pro-IL-1β into its mature form by caspase-1. Mature IL-1β is released extracellularly, binds to its receptor, and initiates a signaling cascade, enhancing TGF-β1 production, promoting CFs activation, and inducing ECM synthesis, ultimately contributing to cardiac fibrosis. Abbreviations: SARS-CoV-2: severe acute respiratory syndrome coronary virus 2, CFs: cardiac fibroblasts, ECM: extracellular matrix, EMT: epithelial-mesenchymal transition, NLRP3: NLR family pyrin domain containing 3, ACE2: angiotensin converting enzyme 2, NF-κB: nuclear factor kappa-light-chain-enhancer of activated B cells, ROS: reactive oxygen species, IL-1β: Interleukin 1 beta.

In conclusion, our study sheds insights on the molecular mechanisms underlying the effects of the S1 protein on CFs and the development of cardiac fibrosis. As illustrated in Figure 8, the S1 protein binds to ACE2, activating intracellular NF-κB signaling. Accumulation of ROS triggers NLRP3 inflammasome activation and IL-1β production. Subsequently, IL-1β binds to its receptors, initiating signaling cascades that upregulate the transcription of TGF-β1, ultimately leading to cardiac fibrosis.

## Data Availability

Data are available upon request.

## Supplementary Materials

The following supporting information can be downloaded at: https://www.mdpi.com/article/10.3390/cells13161331/s1, Figure S1: Effect of S1 protein on CFs mitochondrial morphology; Figure S2: Effect of S1 protein on CFs mitochondrial calcium levels; Figure S3: Effect of S1 protein on CFs mitochondrial ROS production.
