# Supplementary material for: Spike Protein of SARS-CoV-2 Activates Cardiac Fibrogenesis through NLRP3 Inflammasomes and NF-κB Signaling
Source: Cells. 2024 Aug 11;13(16):1331. doi: 10.3390/cells13161331 (PMC11353017; doi:10.3390/cells13161331)
Supplement: Supplementary file 1 [file cells-13-01331-s001.zip › cells-3089260-supplementary.pdf]

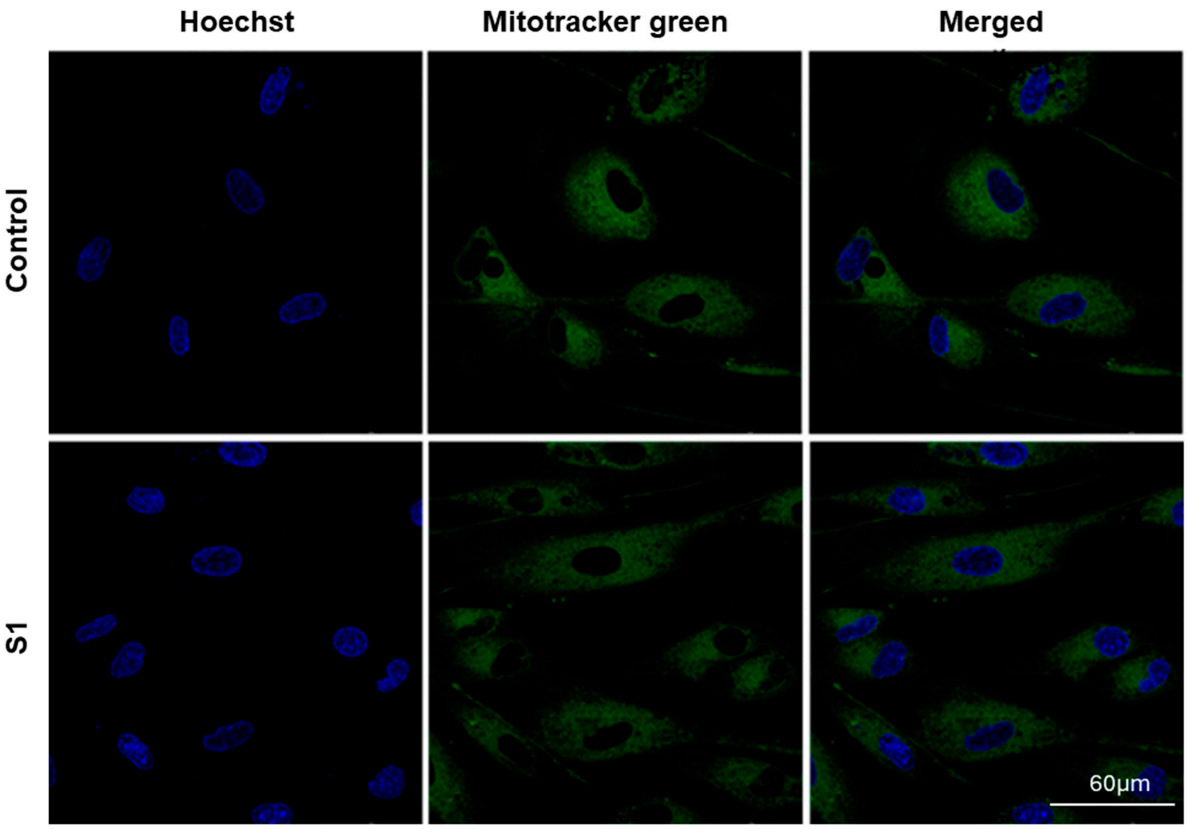

Figure S1. Effect of S1 protein on CFs mitochondrial morphology. S1 protein did not change the morphology of mitochondria in CFs after 24 h of treatment.

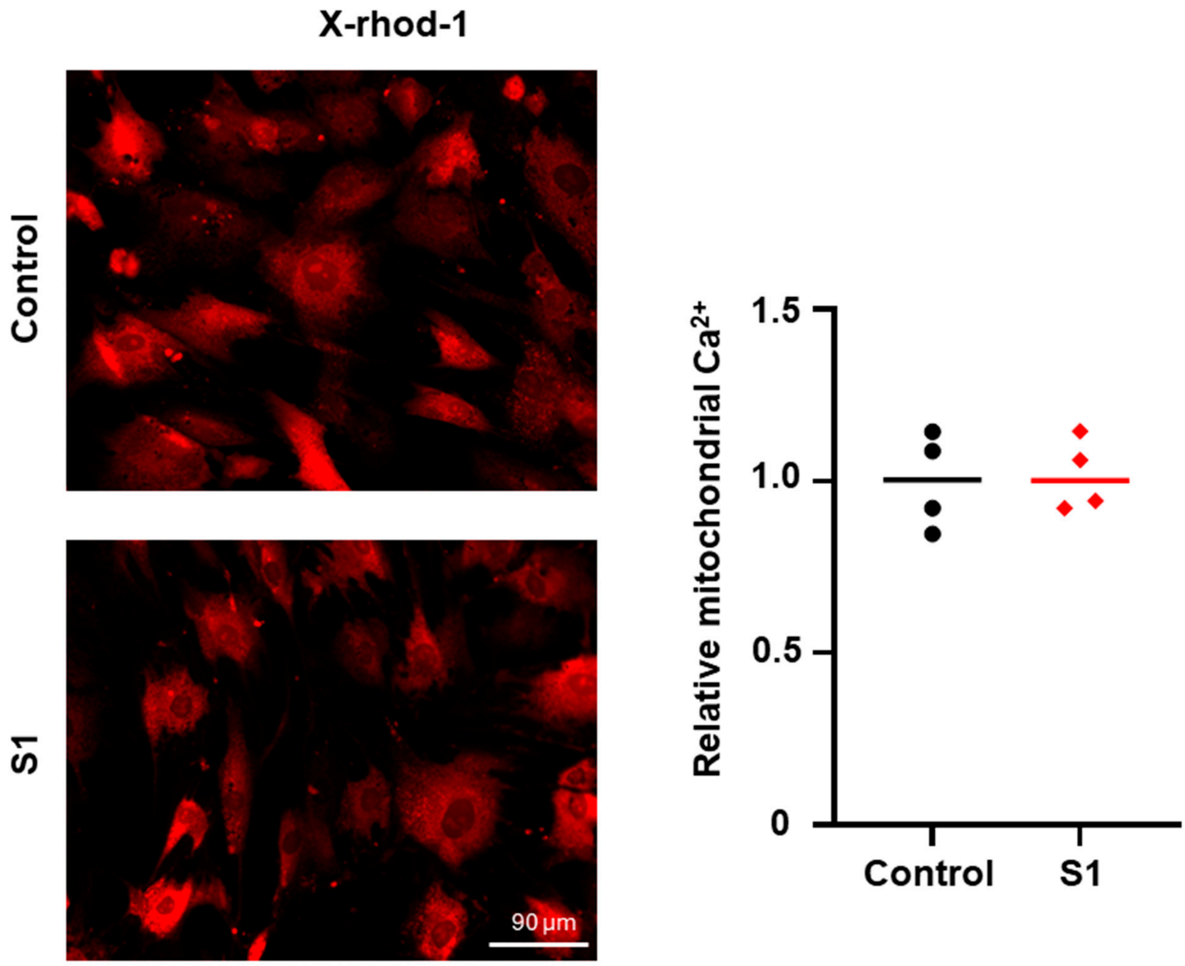

Figure S2. Effect of S1 protein on CFs mitochondrial calcium levels. S1 protein did not change mitochondrial calcium content in CFs, as assayed by X-Rhod-1 staining for mitochondrial calcium (n = 4 independent experiments).

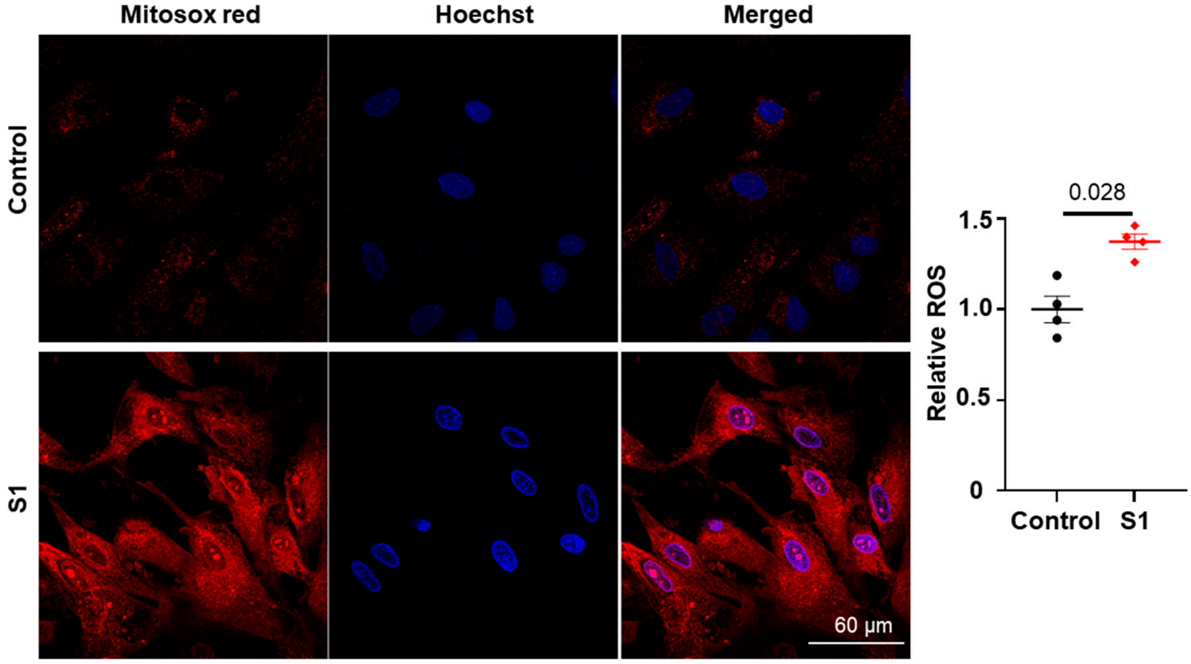

Figure S3. Effect of S1 protein on CFs mitochondrial ROS production. S1 protein increased mitochondrial ROS levels in CFs, as assayed by MitoSOX red staining for mitochondrial ROS (n = 4 independent experiments).
